# Current Strategies for Tracheal Decellularization: A Systematic Review

**DOI:** 10.1155/2024/3355239

**Published:** 2024-02-06

**Authors:** Dhihintia Jiwangga, Ferdiansyah Mahyudin, Gondo Mastutik, Juliana, Estya Nadya Meitavany

**Affiliations:** ^1^Doctoral Program of Medical Science, Faculty of Medicine, Universitas Airlangga, Surabaya, Indonesia; ^2^Department of Orthopaedic and Traumatology, Faculty of Medicine, Universitas Airlangga, Dr. Soetomo General Academic Hospital, Surabaya, Indonesia; ^3^Department of Anatomic Pathology, Faculty of Medicine, Universitas Airlangga, Surabaya, Indonesia; ^4^Faculty of Medicine, Universitas Airlangga, Surabaya, Indonesia; ^5^School of Biomedical Engineering and Imaging Sciences (BMEIS), King's College London, London, UK

## Abstract

The process of decellularization is crucial for producing a substitute for the absent tracheal segment, and the choice of agents and methods significantly influences the outcomes. This paper aims to systematically review the efficacy of diverse tracheal decellularization agents and methods using the PRISMA flowchart. Inclusion criteria encompassed experimental studies published between 2018 and 2023, written in English, and detailing outcomes related to histopathological anatomy, DNA quantification, ECM evaluation, and biomechanical characteristics. Exclusion criteria involved studies related to 3D printing, biomaterials, and partial decellularization. A comprehensive search on PubMed, NCBI, and ScienceDirect yielded 17 relevant literatures. The integration of various agents and methods has proven effective in the process of tracheal decellularization, highlighting the distinct advantages and drawbacks associated with each agent and method.

## 1. Introduction

Trachea's abnormality has been a rising problem in recent years, both congenital abnormalities and acquired abnormalities. Congenital defects may vary in degree and forms, including tracheal agenesis, massive tracheo-oesophageal fistula, and/or tracheomalacia. Acquired defects are commonly caused by trauma, be it blunt or sharp. According to literature, the worst and the most common case of traumatic tracheal abnormalities is post intubation tracheal stenosis (PITS), with the prevalence of 6%–21% of intubated patients. Around 10% of mild stenosis patients may remain undetected for more than 10 years. The newest literature shows that the prevalence of PITS in London is 926 new cases per year [1].

There are 2 types of tracheal repair based on the affected segments. In short-segment defect, which is defined as defects in less than half of the total tracheal length in adults and/or less than a third of the total tracheal length in children, the go-to procedure would be either an end-to-end tracheoplasty or a slide tracheoplasty [2]. On the other hand, long-segment defect is defined as defects in half or more of the total tracheal length in adults and/or a third or more of the total tracheal length in children. There are still no definitive treatments for long segment defects. The patient would usually receive temporary palliative care, such as T-tubes or stents, which have a high rehospitalization and infection prevalence [2].

In recent years, the field of regeneration medicine has been trying to develop a definitive treatment for long-segment tracheal defects and the most promising method right now would be replacing the affected part. Tracheal grafts can be divided into groups such as autologous, homograft, prosthetic, and combined graft. Each of the groups has their own strengths and weaknesses. In autologous and homografts, the problem lies in finding a donor, vascularization failure, failure in thriving, and the need for prolonged immunosuppressants. Prosthetic grafts on the other hand may be a solution to some of those problems. However, prosthetic grafts are too inflexible and proinflammatory, making them the second-best option for a tracheal graft [3].

In order to find a suitable tracheal replacement, graft materials development has been increasing these past few years. Tissue engineering has been used in various ways, such as blood vessels and heart valves. Tissue engineering needs 3 major components, which are scaffolds, healthy cells, and a bioreactor.

Scaffolds are the base of the new cells. Scaffold should have the same mechanical properties of the original organs and should be able to support the cells' adhesion, migration, proliferation, and differentiation. According to these standards, the best tracheal scaffold right now would be a decellularized trachea as it has the same biomechanics, flexibility, and proangiogenic. The purpose of decellularization is to remove the immunogenic components of the hosts without harming the extracellular matrices (ECMs) since ECMs' main function is to grow, maintain, and regenerate the cells. ECM's main components are collagen, proteoglycan, glycoprotein, and glycosaminoglycan. There are 3 methods of decellularization, which are enzymatic, chemical, and physical. The most popular one is the chemical decellularization method where detergent is used to decellularize the scaffold. In tracheal decellularization, the tricky part is the cartilage as it is really thick and will take time for the detergent to penetrate; meanwhile, the ECMs cannot take that much detergent exposure. Thus, this paper is written to evaluate the various decellularization methods and to find one that removes the most immunogenic components yet preserve the most ECMs [4, 5].

The objective of this research is to assess the effectiveness of various tracheal decellularization methods in experimental animals' trachea by the deoxyribonucleic acid (DNA) count and histopathological analysis (HPA) examinations.

## 2. Materials and Methods

**Figure 1:**
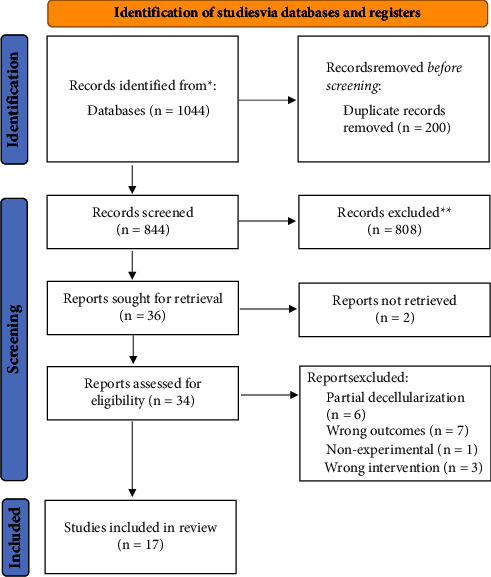
Flowchart of the data gathering process.

The inclusion criteria for this research were animal experimental studies published between the years 2018 and 2023, written in English, primarily studying the process of tracheal decellularization, and describing the outcomes of histopathological analysis, DNA, ECM, and biomechanical characteristics. Excluded studies were studies aiming for partial decellularization, having end products of nontracheal grafts, and/or involving 3D printing and biomaterials. The search was done through PubMed, NCBI, and ScienceDirect using the terms trachea or tracheal and decellularization or decellularization. The last search was done in September 2023. The population, intervention, comparison, and outcome (PICO) framework was used as described in Table 1. The detail of the literature identification process was explained using the PRISMA flowchart in Figure 1.

## 3. Results and Discussion

A comprehensive search of PubMed, NCBI, and ScienceDirect yielded 1044 potentially eligible studies. Of these, 200 duplicates were removed. Upon title and abstract screening, 808 studies were excluded due to being reviews, case reports, editorial comments, not in English, or unrelated to the topic. Two studies were disqualified due to unavailability of full texts. Subsequently, a meticulous full-text review led to the exclusion of 16 more studies, including those with partial decellularization, improper outcomes, incorrect interventions, and nonexperimental designs. Consequently, a total of 17 studies were selected for inclusion in this review. The characteristics of the methods, agents used, and outcomes of each included studies are presented inTable 2.

### 3.1. Tracheal Anatomical Properties

Structurally, the tracheobronchial system can be classified into the conductive part (cartilage) and the airway part (noncartilage). It is located in the medial side of the body where it extends from the neck and to the thorax; topographically, it starts from vertebrae C5-6 and extends down until T5 where it will branch into 2 bronchi. It connects the larynx and the bronchus, and functionally, it is semiflexible, 1.5–2 cm wide, and 10−13 cm long [3, 22].

Tracheal structure includes mucosa, submucosa, hyaline cartilage, and adventitial layer. The mucosal layer includes pseudostratified columnar epithelium and goblet cells; goblet cells will secrete mucous to trap the debris and dirt for the cilia will sweep them away. Submucosa is the deepest part of the tracheal lumen with the most blood vessels and nerve, and its function is to maintain tracheal structural integrity [3, 22].

### 3.2. Tracheal ECM Roles

Tracheal biomechanical properties come from the ECMs which consist of glycosaminoglycan (GAG), collagen, proteoglycan, and other glycoproteins. Collagen is the main component that gives the trachea its biomechanical properties. The collagen fibers make the trachea characteristically laterally rigid and longitudinally flexible. In addition, the ECMs have three main functions, which are intercellular signalling via paracrine signalling, intracellular signalling via autocrine signalling, and cellular formation via mechanical pressure [3, 22, 23].

### 3.3. Tracheal Tissue Engineering

Tracheal tissue engineering has been on the rise due to the complications of autografts and allografts. Tracheal tissue engineering includes resecting the affected organ and changing it with a scaffold that has been seeded with stem cells. The main components of tracheal tissue engineering are the scaffold, cell source, agent, and method [3, 22, 23].

### 3.4. Tracheal Scaffold

There are two main types of tracheal scaffolds with their own strength and weaknesses. The first is the synthetic tracheal scaffold. It is more versatile when it comes to shape and size, but the macro- and microanatomy of the scaffold is lacking compared to the biological scaffold. One of the examples of the synthetic tracheal scaffold includes biodegraded molecules from polyglycolic acid and nanocomposite polymer (POSS) covalently bonded to polyurethane (PCU) [24].

The second type is the biological decellularized scaffold. This type is more popular and favourable since it supports the cellular adhesion, proliferation, and differentiation process [25]. The decellularization process is needed in order to lose all the immune-inducing systems within the trachea that can be activated with major histocompatibility complex I and II (MHC-I and MHC-II) [23]. The components of the natural decellularized scaffolds are exactly like the original. The only downside is that during the decellularization process, there might be some cellular and structural changes due to ECM destruction. Therefore, some researchers are looking for a way to minimize the ECM destruction while optimizing the decellularization process. One of the advantages of using bioscaffold is that the patients have no need of taking immunosuppressants since the tracheas are decellularized and seeded with the patient's stem cells [22].

### 3.5. Decellularization Process

Decellularization is the act of eliminating immunogenic cells without damaging the ECMs; ECMs here refer to structural protein (collagen and elastin), special protein (fibrillin, fibronectin, and laminin), proteoglycan (heparin sulfate, chondroitin sulfate, keratin sulfate, and GAG), and growth factors. The advantages of decellularized tracheas are less antigenicity, inflammation, and graft rejection [26].

Every decellularization process needs a decellularization agent and every decellularization agent has its own pros and cons. Some of the popular decellularization agents used are mentioned in the following.

#### 3.5.1. Chemical Agent

The types of chemical agents commonly used include acids, bases, detergents, hypotonic-hypertonic solutions, and solvents to lyse and kill cells [26, 27]. Some of the chemicals used in the decellularization process are as follows.

*(1) Acid and Bases*. Decellularization methods involving acids and bases catalyse the hydrolytic degradation of biomolecules, cytoplasmic components, and nucleic acids [28]. Like detergents, they have the capacity to disrupt the extracellular matrix (ECM) constituents and structures. Acidic compounds either donate hydrogen ions (H+) or form covalent bonds with electron pairs to facilitate hydrolytic degradation. Peracetic acid (PAA), hydrochloric acid, and acetic acid are commonly employed for the decellularization process [29, 30].

Peracetic acid and hydrochloric acid are among the acid agents employed in the decellularization process. Peracetic acid functions by disrupting cell membranes and solubilizing cytoplasmic organelles. However, it comes with the drawback of damaging the extracellular matrix (ECM) architecture. On the other hand, hydrochloric acid induces cell lysis, denatures proteins, and catalyses the hydrolytic degradation of biomolecules. Yet, its disadvantage lies in its impact on intracellular molecules, particularly glycosaminoglycans (GAG) [31–33]. Hence, it is essential to choose suitable acids and concentrations. Peracetic acid (PAA) at 0.1% concentration is considered an optimal treatment for thin tissues, as it minimally affects extracellular matrix (ECM) structures and components [34].

In contrast to acidic compounds, alkaline substances can release hydroxide ions (OH−) and interact with acids to produce salts. Ammonium hydroxide, sodium hydroxide, and sodium sulphide are commonly used bases in decellularization [35]. Bases achieve tissue decellularization by denaturing chromosomal DNA and inducing cellular lysis. Particularly, alkaline solutions with a pH exceeding 11 prove effective in eliminating cellular remnants, given the susceptibility of DNA to denaturation [34].

Ammonium hydroxide functions by solubilizing cytoplasmic components, disrupting nucleic acids, and catalysing the hydrolytic degradation of biomolecules. However, drawbacks include its impact on the GAG content, collagen, and growth factors, as well as a weakening of the mechanical properties of the scaffold. Alkaline solutions with a pH range of 10–12 can cause significant harm to collagen fibers, fibronectin, and GAGs. In addition, they may trigger intense host responses and lead to the formation of fibrotic tissues [32, 35, 36].

*(2) Organic Diluent*. The mechanism of action of these agents involves cell membrane lysis. Commonly utilized types include alcohol, acetone, and 1% tributyl phosphate (TBP) for solid tissue decellularization. In the case of acetone and alcohol, they have the capacity to precipitate ECM proteins and influence ECM ultrastructure. In the decellularization of solid tissues such as tendons, tributyl phosphate is more effective at preserving ECM structure and composition. In addition, TBP demonstrates virucidal effects (inactivating viruses) without affecting coagulation factors [26, 27].

*(3) Hyper/Hypotonic Fluid*. Hypotonic solutions lyse cell membranes by increasing cell volume beyond their limits. These agents do not significantly impact changes in ECM components. Hypertonic solutions cause cells to lose volume and eventually die. The drawback of this type is its incapacity to effectively remove residual DNA from cell death. In the process, scaffold materials are immersed in hypotonic and/or hypertonic solutions over several cycles to achieve optimal results [26, 27].

**Figure 2:**
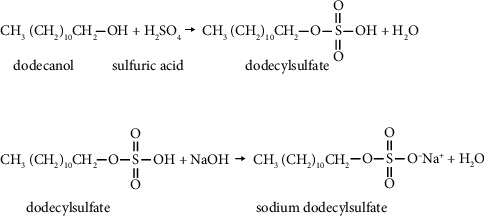
The chemical reaction for sodium dodecyl sulfate.

*(4) Ionic Detergent*. Ionic detergent includes sodium dodecyl sulfate (SDS), sodium dodecyl cholate (SDC), Triton X-200, and sodium hypochlorite. The most popular one right now is the SDS 0.1%. Sodium dodecyl sulfate (SDS), also known as sodium lauryl sulfate (SLS), is a widely recognized anionic surfactant with amphiphilic properties, combining hydrophilic and hydrophobic characteristics. It dissolves lipids but can cause skin and eye irritation. SDS is produced by reacting dodecanol with sulfuric acid and sodium hydroxide, and the chemical reaction produced is as follows (Figure 2) [37, 38].

SDS 0.1% is a commonly used agent, but various concentrations of SDS (0.01, 0.025, 0.05, 0.075, and 0.1%) have started to be studied in the context of the decellularization process. Like nonionic detergents, this agent can also influence the ECM structure. Furthermore, this agent can remove growth factors in the ECM [26, 27].

*(5) Nonionic Detergent.* Nonionic detergent will disrupt the cellular structure by destroying the lipid-lipid and lipid-protein bind without destroying the protein-protein compound. Triton X-100 0.5% is the most popular one due to its ability to maintain protein structures and GAG sulfate. The advantage of these agents is that they do not disrupt the bonds between proteins or sulfated glycosaminoglycans. Their drawback, however, is their potential to reduce the concentration of laminin/fibronectin in the ECM structure [26, 27].

*(6) Zwitterionic Detergent*. This agent has the combined properties of ionic and nonionic detergent. This agent is usually used for mild-moderate decellularization. Some of the examples include sulfobetaine-10 (SB10), sulfobetaine-16 (SB16), and 3-[(3-cholamidopropyl) dimethylammonio]-1-propane sulfonate (CHAPS) [26, 27].

#### 3.5.2. Physical Agent

Physical agents employ various physical conditions, including temperature, mechanical force, pressure, and electrical currents, to disrupt cell membranes and induce lysis, ultimately resulting in the removal of cells from the scaffold matrix. These physical methods represent an alternative approach to decellularization and have been explored for their potential benefits in tissue engineering [26, 27].

*(1) Freezing-Thawing*. Decellularization is done by purposefully freezing the intracellular fluid and destroying the cells; the downsides of this method are that it cannot get rid of the genetic materials and ruins the ECMs. This agent is usually done with the help of some detergents and/or nuclease to optimize the results [26, 27].

*(2) Mechanical Pressure*. These agents are typically employed in organs or tissues with less dense ECM (e.g., the lungs and the liver). Their mechanism of action involves releasing cells from the tissue or organ through applied pressure. However, one drawback of these agents is their potential to cause structural damage to the ECM. Precisely controlling the applied force is a crucial aspect of using these agents effectively [26, 27].

*(3) Electroporation*. Another term for this agent is nonthermal irreversible electroporation. Its mechanism involves disrupting the potential difference across the cell membrane, thereby interfering with cell permeability and ultimately causing cell death using an electric current. An advantage of this agent is its ability to preserve the biomechanical structure of the ECM. However, a limitation is its suitability for use in small, thin tissues or smaller organs [26, 27].

*(4) Immersion and Agitation*. Immersion and agitation represent a more suitable approach for small, delicate, and thin organ sections, as well as tissues lacking intrinsic vascular structures [39–41]. Immersion and agitation involve submerging tissues in decellularization solutions with continuous mechanical agitation, and its effectiveness relies on various parameters such as agitation intensity, decellularization agent, and tissue dimensions [39, 42]. The process, following tissue immersion in the agents, facilitates cell rupture, cell detachment from basement membranes, and the elimination of cellular components. Employing immersion and agitation as an optimal physical decellularization method offers numerous advantages. First, dynamic immersion and agitation achieve a more uniform detergent exposure compared to static decellularization, resulting in better decellularization outcomes with reduced exposure time to aggressive agents [43, 44]. Second, this method minimally impacts ECM surface structure, collagen integrity, mechanical strength, and GAG content [44–48]. Third, it is more accessible and easily executed than whole organ perfusion. However, it may inflict more tissue damage compared to perfusion due to the limited chemical diffusion caused by agitation [34].

*(5) Sonication*. Sonication operates by generating acoustic cavitation bubbles, inducing shear stress effects, and consequently rupturing the cell membrane. It facilitates agent penetration by emitting vibrations, aiding in the removal of cellular debris. However, the drawback lies in the potential disruption of main structural fibers and adverse effects on vascular tissues with high power or prolonged duration of sonication [49–51].

#### 3.5.3. Enzymatic Agent

*(1) Trypsin*. The working mechanism of this agent involves breaking the peptide bonds between carboxyl, arginine, and lysine. Several studies using 0.5% and 1% trypsin with exposure times of 48 and 24 hours have been shown to cause ECM damage. Further research on 0.02% trypsin for 1 hour has a less significant impact on the ECM structure after decellularization [26, 27].

*(2) Exo/Endonuclease*. The working mechanism of this agent involves breaking the bonds of RNA and DNA components. Commonly used types of this agent include DNase (0.2–0.5 mg/mL) and RNase (0.2–50 *μ*g/mL). The application of this agent is often combined with others to remove any remaining DNA/RNA from the decellularized scaffold [26, 27].

*(3) Dispase*. The mechanism of action of this agent involves catalysing primarily collagen IV and fibronectin in the basement membrane, separating it from the epithelial layer. Commonly used types of this agent include 4 mg/mL Dispase II for 45 minutes to remove it. For the removal of hair, fat, and epidermis, Dispase II at 0.24 mg/mL for 3 hours is used [26, 27].

*(4) Phospholipase A2*. The mechanism of action of this agent involves damaging phospholipid components. This agent is typically used in combination with other decellularization agents. Its advantage is in preserving collagen and proteoglycans in the ECM structure although it has a minor impact on GAG composition [26, 27].

#### 3.5.4. Compound Agent

Physical, chemical, and enzymatic agents each have their own advantages and disadvantages and work through distinct mechanisms. To make the decellularization process more effective and efficient, the use of combinations of agents has been explored. For example, a combination of physical and chemical methods has led to the development of cryochemical agents for liver decellularization. Another example involves the combination of agitation, alkali, detergent, enzymes, and light-emitting diodes in the decellularization process of tracheal organs [26, 27].

### 3.6. Decellularization Methods

After determining the decellularization agent, the next step would be choosing the method. The organ's or tissue's characteristics need to be considered in choosing the decellularization method. Some of the examples include the following.

#### 3.6.1. Whole Organ Perfusion

It is used on large and dense organs or tissues with internal vascularization and usually uses ante- or retrograde perfusion. This method uses the organ's own vascular system to distribute the decellularization agent; after distributing the decellularization agent, the dead cells and the remaining decellularization agent will be drained through the veins. Some of the organs that can be decellularized through this method are the muscle, lungs, liver, kidneys, and heart [26, 27].

#### 3.6.2. Immersion and Agitation

This method is used for organs without any decent internal vascularization; the tissue would be immersed and agitated in the decellularization agent where the agent will diffuse into the cells. The factors that affect the outcome include the agitation intensity, decellularization agent, and tissue density and size. It usually takes 1-2 hours for thin preparations and 12–72 hours for thick preparations to finish. DNase and/or RNase are needed to clean out the remaining cellular components. The downside of this method is that some of the cells could already be destroyed via DNase and/or RNase before even coming into contact with the decellularization agent which will affect the ECM's integrity [26, 27].

#### 3.6.3. Pressure Gradient

Pressure gradient is commonly used on hollow organs. This mechanism is similar to immersion and agitation. However, it is more optimized by creating a pressure gradient between the extracellular space and the intracellular space, thus optimizing the diffusion process [26, 27].

#### 3.6.4. Supercritical Fluid

This method uses a highly viscous and transportable fluid to kill the cells. This mechanism can also preserve the sample and minimalize the lyophilization process [26, 27].

### 3.7. Post-Decellularization Evaluation

Post-decellularization evaluation is used to assess the decellularized organs; some of the parameters being measured are the number of DNA strains left, the toxicity, the cellular immunity, and the ECMs [27]. Some of the evaluation methods are discussed as follows.

#### 3.7.1. Histologic and Immunohistochemistry (IHC) Evaluation

Histologic and IHC evaluations are mainly qualitative testing, yet also may serve as a quantitative examination. Qualitative testing is done by comparing the quality of the sample to the original organ, while quantitative testing is done by counting the number of cellular nuclei. First, the organ would need to be fixated in a paraffin block and then cut into smaller pieces. Subsequently, the samples are stained with hematoxylin and eosin for differentiating the ECMs and the nuclei [20, 52]. For a more focused ECM examination, the staining used are toluidine blue and safranin O for GAG assessment, Masson's trichrome for collagen, and Van Gieson for elastin [20, 53]. IHC examination is used to assess the scaffold's remaining immunological factors [54, 55]. Other than that, IHC examinations can also evaluate the vascularization potential of the tissue by using anti-CD-31, anti-vWF, and anti-FGF [54]. The downside of this method is that it takes a long time and depends heavily on the examiner [56].

#### 3.7.2. DNA Quantifications

DNA quantification shows the number of DNA in the decellularized graft as a marker for the graft's immunogenicity ability. The recipient's immunogenicity tolerance is <50 ng dsDNA/mg of the graft's dry weight; this number is also the marker of a successful decellularization process. The first step of DNA quantification is to put the sample into an enzymatic solution usually phosphate buffer saline (PBS) for around 24 hours and then the DNA is isolated and examined in a special machine. There are 2 types of DNA quantifier; one of the machine examples is the Quant fluor dsDNA System E2670 Promega that shows the results in DNA/mg scaffold's dry weight and a nanophotometer that shows the results in nanogram [21].

#### 3.7.3. GAG Quantifications

GAG quantification is used to measure a method's effectivity in clearing out the cells without destroying the ECMs, thus the need for a control sample for comparison. The GAG quantification is done using a light spectrophotometer [21].

#### 3.7.4. Biomechanics Testing

Biomechanics testing is also quantitative testing that the decellularized trachea is being compared to the control samples. The tracheas will be pulled from 2 sides uniaxially. The data consist of the force given and the increase in length. The proximal, intermediate, and distal parts of the trachea have different biomechanics; thus, testing all 3 of them is recommended [52].

#### 3.7.5. Toxicity Testing

Toxicity testing is done to check on the scaffold's toxicity level post-decellularization and sterilizing. The purpose is to assess the toxicity level of the tissue induced by the decellularization agents and/or the remaining bacteria on the tissue; thus, toxicity testing is usually done alongside a bacterial load examination to find out if the problem is in the sterilizing process or the decellularization agents [20, 27, 57].

In Table 2, the DNA counts and staining results of various tracheal decellularization methods from 2018 to September 2023 are presented.

## 4. Conclusions

In conclusion, the selection of a decellularization agent and method should be carefully tailored to the specific tissue or organ under consideration, as each comes with its own set of advantages and disadvantages. According to the findings from the reviewed studies, the optimal scaffold with the minimal DNA content and preserved extracellular matrices (ECMs) is achieved by combining various agents of physical, chemical, and enzymatic nature.

Nonetheless, it is essential to recognize that the quest for the ultimate decellularization method is an ongoing process. Further experiments and research are imperative to explore and refine the selection of agents and methods, aiming for the development of the most effective, safe, and versatile decellularization protocol. The study's limitations encompass the need for more extensive investigations across various tissue types, focusing on the effects of decellularization on the seeding process and its potential immunogenic effects on the recipient organ. Future research may involve comparative analyses of different decellularization techniques, shedding light on their impacts on tissue biomechanics and immunogenicity, to further advance the field of tissue engineering.

## Figures and Tables

**Table 1:** PICO framework description used in this study.

Components	Description
Population	Experimental animals
Intervention	Decellularization
Comparison	Various decellularization methods
Outcome	Histopathological analysis, DNA quantification, ECM evaluation, biomechanical analysis

**Table 2:** Table of protocols of tracheal decellularization and their results.

Authors	Donor	Methods and agents used	Outcomes
Martínez-Hernández et al. [6]	Rabbit	PBS + SDS 2% + penicillin-streptomycin 5% + amphotericin B 5% for 5 weeks under continual stirring	(1) HE: evaluating the decellularization process showed minimal chondrocyte debris in cartilage, and DNA quantification did not detect values >50 ng (almost 100% cell removal compared to fresh trachea) (2) Biomechanical test: using the tensile test and the radial compression test showed reduced biomechanical properties vs. native trachea

Wang et al. [7]	Pig	Group A: control group Group B: SDS decellularized only Group C: −80°C storage for 3 months only Group D: −80°C storage for 3 months and then decellularized Group E: after decellularization and then put into −80°C storage Decellularization solution: SDS 3%	(1) HE: group C still retained lots of nuclei, and groups B, D, and E have removed tracheal columnar epithelium cells and mucosal and submucosal, but ECM matrix still existed (2) MT: groups B, D, and E have removed epithelial layer, and all groups have enough ECM matrix (3) DAPI: compared with the control group, the experimental group (groups B, C, D, and E) did not significantly affect the cell nucleus in cartilage tissue (4) Safranin-O: there was no significant difference in the staining of the tracheal cartilage in the cryopreservation group compared with the control group (5) DNA quantification: group E (33.66 ng/mg), D (53.16), B (110.33), C (306.83), A (421 ng/mg) (6) Biomechanical test: decreased compressive strength in group E; tensile strength for all groups still good (within 50% tensile displacement, no fracture was found and the shape was preserved)

Batioglu-Karaaltin et al. [8]	New Zealand rabbits	Lyophilization and DEM combination Group 1: LYP Group 2: LYP + DNase (150 U/mL) + MgSO_4_ (50 mmol) + LYP Group 3: LYP + deoxycholic acid + DNase + MgSO_4_ + LYP Group 4: LYP + deoxycholic acid + Triton X-100 + DNase + MgSO_4_ + LYP Group 5: LYP + SDS+1% (w/v) Triton X-100 + LYP Group 6: LYP + SDS + PBS + DNase + LYP Group 7: control: untreated trachea	(1) DNA concentration: <50 ng/mg (2) GAG: the cartilage matrix significantly decreased after decellularization and significantly different except group 3 (3) HE: group 1 with most cells retained, same with 4 and 6 (4) SEM: no cellular components on luminal surface of groups 2 and 6 and preserved cartilage matrix in group 6 (5) Biomechanical test: no different with the normal trachea but rigidities groups 1, 3, and 5 were higher

Greaney et al. [9]	Male WT Sprague–Dawley rats (*Rattus norvegicus)*	Decell A method: (i) Antibiotic rinse (ii) Perfused 50 ml/min: 30 min 0.0035% Triton X; 10 min 1M NaCl, 30 min PBS, 1.5 h 0.1% SDC, 30 min PBS, 1 hr 15 IU/ml benzonase in buffer, 30 min PBS, 10 min 0.5% Triton X, 4 × 20 min PBS Decell B method: (i) Antibiotic rinse (ii) 25x cycles perfused 50 ml/min: 4 h 4% SDC, 3 × 5 min diH_2_O, 3 h 2000 kU DNase-l in 1M NaCl, 2 × 5 min diH_2_O (iii) Store in PBS at 4C between cycles	(1) Cellular removal method B is harsher; complete cellular removal but DNA quantification in both reaches the goals but method A is better since it removes epithelial and stromal cells, while leaving the cartilage intact, where B removes all including chondrocytes (2) Matrix ECM and collagen are not well preserved in both methods (3) Mechanical structure: stiffer in both groups (4) Decell A produced a stiffer tissue, probably due to intact engaged collagen I in the setting of reduced collagen III. In contrast, Decell B resulted in a tissue with severely compromised ECM structure, including damage to collagen I fibers

Stocco et al. [10]	Pigs	Group 1: cryopreservation Group 2: tracheal decell using physical + enzymatic + chemical (i): Freezing and lyophilization for 12 h (ii): dH_2_O + 0.05% trypsin +0.02% EDTA enzymatic solution (iii): Washing w 2% Tergitol detergent solution + 0.8% NH_3_OH in dH_2_O (iv) Washing w dH_2_O repeated for 12 cycles and then frozen at −20°C and lyophilized	(1) DAPI and DNA quantification: show reduced number of cell and DNA content (2) HE: reduction in CryoT, no cells were detected in Decell T (3) Alcian blue staining: both group still has retained cytoplasmic granules and GAG, less intense in Decell T; however, 56.4% GAG in group B were maintained (4) Masson's trichrome: all collagen remained intact in all (5) SEM: some cells were still recognizable in CryoT; complete removal of epithelium in decellT, both displayed organized collagen fiber

Sun et al. [11]	Rabbit	Tracheae were incubated in a detergent solution containing 0.25% Triton X-100 (Biofroxx, Einhausen, Germany) and 0.25% sodium deoxycholate (Sigma, California, USA) at 37°C for 24 h under vacuum. The scaffolds were then washed in sterile distilled water three times for 30 min and incubated for another 24 h in sterile distilled water at 4°C. Following the wash step, the scaffolds were subjected to enzymatic digestion with 2 kU/mL DNAse (Sigma, California, USA) and 4 U/mL RNAse (Biofroxx, Einhausen, Germany) in 1 M NaCl at 37°C at different times (8 h (VAD 8 h group), 16 h (VAD 16 h group), and 24 h (VAD 24 h group)) as experimental groups. Finally, the decellularized tracheal segments were stored in 4°C PBS containing 1% antibiotic and antimycotic solution	(1) HE: 24 h group showed the least cell nuclei (2) MT: all groups show similar structural integrity (3) Alcian blue: all groups show reduced GAG expression

Hong et al. [12]	Rabbit	(1) Hypotonic 10 mM tris buffer containing a serine protease inhibitor (5% phenylmethylsulfonylfluoride in ethanol, 0.35 mL/L), a metalloprotease inhibitor (5 mM ethylenediaminetetraacetic acid (EDTA)), and 5 mL/L penicillin/streptomycin solution (10,000 U/mL/10,000 mg/mL) for 36 h at room temperature (2) High saline (1.5 M potassium chloride), 50 mM tris buffer containing 1% Triton X-100 (octyl phenoxy polyethoxyethanol), as well as protease inhibitors and antibiotics for 48 h at room temperature (3) Rinsed with Hanks' physiological buffer and then submerged for 5 h at 37°C in Hanks' buffer containing 90 U/mL deoxyribonuclease (type II from bovine pancreas) and 85 mg/mL ribonuclease (type III A from bovine pancreas). In the fourth stage of the decellularization process, the three protocols differ. Samples were immersed in a 1% solution of either (i) sodium dodecyl sulfate (lauryl sulfate, SDS) identified as Triton-SDS, (ii) Triton X-100 identified as Triton-Triton, or (iii) tributyl phosphate (TnBP) identified as Triton-TnBP. This second wash was carried out with 50 mM Trizma's base and antibiotics for 48 h at room temperature. Samples were subsequently rinsed with distilled water and then in a second modification of the process, immersed in a 50 mM tris buffer adjusted to pH 9.0 with antibiotics. Specimens remained in the pH 9.0 wash for 24 h at room temperature to remove residual surfactant before being washed in phosphate buffered saline (PBS) containing antibiotics for 24 h	(1) Overall, the DNA content was reduced by an average of 97.14% (fresh tissue mean DNA, 1857.59 ± 98.70 ng/mg and decellularized tissue mean DNA 53.21 ± 17.42 ng/mg; *P* < 0.0001) (2) The GAG content (*n* = 4 per run; *n* = 12 per group) was not significantly affected by the decellularization process (fresh tissue mean GAG 24.96 ± 1.89 *μ*g/mg and decellularized tissue mean GAG 24.17 ± 2.01 *μ*g/mg; *P*=0.6459) (3) H&E revealed that cell nuclei in the perichondrium and the peripheral regions of the ECM were absent in the decellularized tissue and the modulus (fresh tissue mean 13.6 ± 1.8 MPa and decellularized tissue mean 17.3 ± 3.5 MPa; *P*=0.18) and ultimate tensile strength (fresh tissue mean 2.5 ± 0.6 MPa and decellularized tissue mean 3.0 ± 0.4 MPa; *P*=0.29) did not significantly change after decellularization

Baranovskii et al. [13]	Human	In brief, the samples were placed into cryovials that were immersed into liquid nitrogen for 15 minutes, and samples were then thawed in a water bath at 37°C for 30 minutes. This cycle was repeated 5 times.	(1) Safranin-O staining evidenced a certain loss of GAG only in the regions immediately surrounding the pores but a preserved GAG intense matrix in the remaining regions of the LPTCs (2) DNA content of TCs after the decellularization process averaged 35 ± 12 ng/mg. Results showed that endotoxin concentrations were similarly low (less than 1.0 IE/ml) in the conditioned media and control medium

Guimaraes et al. [14]	Porcine	10 cycles of agitation for 48 h with 30 mL of a 2% sodium deoxycholate detergent and ethylenediaminetetraacetic acid (EDTA) 0.02% in an incubator at 36°C and 180 rpm, followed by 3 washings with phosphate-buffered solution (PBS) for 10 min. At the end of this process, the scaffolds were exposed to 30 mL of DNase solution 3 L of DNase +1 mL of 1.3 mM MgSO_4_ and 2 mM CaCl_2_ at 37°C for 12 h under constant agitation	(1) The DNA contents fell from 850 to +123 ng/mg of dry tissue to 20 +8 ng/mg of dry tissue, *P* < 0.001 (2) Cytotoxicity, the cellular viability under the challenge with the homogenate was 42.7% (3) There is an increase in the maximum load and stress in the maximum load in the longitudinal axis, while there was a decrease in the transverse axis. The mechanical properties of the scaffold in PBS solution at 4°C did not show significant differences in the Young modulus, maximum load, and stress at the maximum load (4) There was no significant difference in the percentage of collagen in the trachea before and after decellularization

de Wit et al. [15]	Landrace pigs	Group 1: Supercritical carbon dioxide processed at 37°C with 250 bar for 12 hours as a dynamic run (constant flow of CO_2_) Washed by 25% H_2_O_2_ for 60 min Washed by H_2_O Washed by a 1.25 M NaOH for 30 min Washed by H_2_O pH set at 7.4 ± 0.2 with 0.25 M NaH_2_PO_4_ buffer Group 2: Following the group 1 without NaOH wash Followed by lyophilization Group 3: Following group 2: Subsequently scCO_2_ sterilized with low dose peracetic acid (4.5 mL freshly prepared PAA with 1.25 mL H_2_O, 0.75 mL 25% H_2_O_2_ and 2.5 mL 2 M acetic acid) with 160 bar for 4 h at 35°C as a static run Control group: Treated with Triton X-100/sodium deoxycholate for 24 h under vacuum conditions followed by a 48 h wash step Freeze dried for 3 days under vacuum conditions	(1) HE: method 1 resulted in efficient chondrocyte nuclei removal compared to other methods (2) Gomori's trichrome: methods 2 and 3 and DEM were comparable to native trachea. Methods 2 and 3 resulted in more incomplete washout of cellular remnants compared to method 1 (3) Fibronectin and laminin staining: Laminin and fibronectin were not observed in all groups (4) GAG: GAG was best preserved in method 3 (48.8 ± 5.5 *μ*g/mg; native 41.7 ± 4.7 *μ*g/mg), followed by method 2 (33.1 ± 11.0 *μ*g/mg), and significantly reduced in DEM and method 1 (20.3 ± 1.9 *μ*g/mg tissue; 5.7 ± 3.2 *μ*g/mg) (5) SEM: basement membrane was disrupted due to method 3 while staying intact in other methods (6) Collagen was loss in method 2 and DEM (7) DNA quantification: the least DNA was found in DEM (15.2 ± 9.2 ng/mg), followed by methods 1, 2, and 3 (87.7 ± 59.7 ng/mg, 385.7 ± 116.8 ng/mg, 412.6 ± 193.8 ng/mg) (8) Mechanical properties: method 3 (10.48 ± 0.45 ng/mg) has the most similar properties to native (8.8 ± 0.8 ng/mg), followed by DEM, method 2, and 1 (4.7 ± 1.4 ng/mg; 4.2 ± 1.4 ng/mg; 1.3 ± 0.9 ng/mg)

Wang et al. [16]	New Zealand white rabbits	DEM with adjusted DNase I dose: 2 kU/ml 4 kU/mL 6 kU/mL 8 kU/mL	(1) HE: 8 kU resulted in almost completely removal of cartilage nuclei (2) Masson trichrome: no significant change in each group compared to the natives (3) Alician blue: GAG n the matrix was defected more as DNase I concentration increase (4) DAPI: blue fluorescence got dimmer as the DNase I concentration increased, indicating less residual DNA (5) DNA quantitative: DNA content was significantly less as the DNase I concentration increased (6) IHC: IHC showed significantly reduced expression of MHC-I and MHC-II in all groups compared to native trachea (7) sGAG: all groups had significant loss of sGAG as DNase I concentration increased (8) Type II collagen: only 8 kU/mL DNase I has significant loss of collagen compared to native trachea (9) Biomechanical properties: anticompression properties got weaken as DNase I increased but not significant in group 2 kU/mL (10) SEM: 6 kU resulted in small gaps in the basal membrane, 8 kU resulted in larger crack in basal membrane

Matias et al. [17]	Dogs	SDS 4% + orbital agitation SDS 4% + orbital agitation + SDS 4% and FBS 12% SDSS 4% + orbital agitation + SDS 4$ + vacuum	(1) Method 3 showed superior DNA removal (gDNA = 13.5 ng/mg) (2) Method 3 showed significant ECM preservation (collagens, GAGs, and PGs) compared to other methods

Milian et al. [18]	Porcine	Experiment 1 A: control B: 0.2% Triton X-100 + 0.25% SDS C: 2% Triton X-100 + 0.25% SDS D: 4% SDC Experiment 2: A: control F: 0.5% SDS G: 1% SDS E: 2% SDS H: 4% SDS	(1) HE: groups C and E had superior effect on chondrocyte removal, while B and D had lower effect (2) Collagen and GAGs: groups C and D preserved more compared to groups B and E (3) DNA: groups C, D, and E resulted in significant decrease of DNA content (4) Elastic: groups D and E preserved elastic fiber, resulting in a nonsignificant difference with the control group Experiment 2: (1) HE: group H removed the most cells (2) Collagen: group E lost the most collagen, while other groups had minimal effect on the collagen

Guimaraes et al. [19]	Porcine	Mix of physical-chemical process: (1) Freezing and thawing for 10 cycles (i) Frozen at −80°C for 24 hours (ii) Thawed for 40 min in 36°C water bath (iii) Washed with PBS 3x and exposed to 2% sodium deoxycholate (iv) Shaken in the incubator at 180 rpm, 36°C for a 48-hour cycle (2) DNA extractions (i) DNase solution for 12 hours (ii) Proteinase K for 12 hours (iii) Extraction with phenol chloroform	(1) HE: complete denudation of the epithelium and mixed glands over the cartilage: 20 ± 8 ng/mg (2) Marked by reduction of the DNA content (mean DNA content)

Hong et al. [12]	Rabbit	Multistep chemical process involving: (1) Solution A, pH 8 in room temperature for 12 hours (i) Trizma base (hypotonic solution) (ii) EDTA (metalloprotease inhibitor) (iii) PMSF (serine protease inhibitor) (iv) Pen/strep (2) Solution B, pH 8 in room temperature for 12 hours (i) Triton X-100 (nonionic surfactant) (ii) EDTA (iii) PMSF (iv) KCl (hypertonic solutions) (v) Trizma base (vi) Pen/strep (3) DNase and RNase, pH 7.35, 37°C for 5.5 hours (4) Solution C, pH 9 in room temperature for 12 hours (i) 70% ethanol (ii) Trizma base (iii) TBP (surfactant like solvent) (iv) Pen/strep (5) Solution pH 9 in room temperature for 22 hours (6) 1% paracetic acid in room temperature for 4 hours (7) Phosphate buffer saline in room temperature for 4 hours	(1) HE: cell nuclei in the perichondrium and the peripheral regions of the ECM were absent in the decellularized tissue (2) Masson's trichrome: major histoarchitecture of the trachea remained unchanged following decellularization (3) Overall DNA count reduced by average of 97.14% (mean DNA content: 53.21 ± 17.42 ng/mg)

Dimou et al. [20]	Murine	Chemical process using: (1) CHAPS solution (8 mM CHAPS, 1M NaCl, and 25 mM EDTA diluted at 1 × PBS; pH 8) for 22 hours at room temperature (2) PBS 1× washing for 30 min at 4°C (3) SDS solution (1.8 mM SDS, 1 M NaCl, and 25 mM EDTA diluted at 1× PBS; pH 8) for 22 hours at room temperature (4) PBS 1× washing for 30 min at 4°C (5) Place sample in *α*-MEM/40% FBS at 37 °C for 48 hours All steps were done under constant agitation and repeated 3 times	(1) HE: complete removal of nuclear and cellular materials after three cycles of decellularization compared to native samples (2) Masson's trichrome: sulfated glycosaminoglycans, collagen, and elastin were preserved in decellularized tissue (3) DNA contents post decellularization was significantly lower (4.1 ng DNA/mg dry tissue) compared to native tissue (722.9 ng DNA/mg dry tissue)

Pai et al. [21]	Ferret and murine	Chemical process using: (1) 10 min with 1X DPBS (100 mL) (2) 8 min with 0.25% SDS (w/v) diH_2_O (40 mL) (3) 8 min with diH_2_O (4) 8 min with triton X-100 v/v in diH_2_O (40 mL) (5) 50 min with 1x DPBS (250 mL)	(1) HE: Luminal cells are absent (2) Masson's trichrome: no difference found between the decellularized group and the control group (3) DNA quantification showed that the decellularized trachea's DNA count is significantly lower compared to the control group (350 ng/mg)

*α*-MEM: minimum essential medium; CHAPS: 3-((3-cholamidopropyl)-dimethylammonio)-propane-sulfonate; diH_2_O: deionized water; DNA: deoxyribonucleic acid; DPBS: Dulbecco's phosphate-buffered saline; EDTA: ethylenediaminetetraacetic acid; FBS: fetal bovine serum; HE: hematoxylin and eosin; KCl: potassium chloride; LYP: lyophilization; MgSO_4_: magnesium sulfate; PBS: phosphate-buffered saline; pen/strep: penicillin-streptomycin; PMSF: phenylmethylsulfonyl fluoride; rpm: rotate per minute; SDS: sodium dodecyl sulfate; SEM: scanning electron microscopy.
